# Perspective on Renal Involvement in Antiphospholipid Syndrome: Implications for Diagnosis, Pathogenesis, and Treatment

**DOI:** 10.3390/jcm14103326

**Published:** 2025-05-10

**Authors:** Ariela Hoxha, Dorella Del Prete, Irene Condonato, Francesca K. Martino, Marco Lovisotto, Federico Nalesso, Paolo Simioni

**Affiliations:** 1 Internal Medicine Unit, Thrombotic and Hemorrhagic Center, Department of Medicine—DIMED, University of Padua, Via Giustiniani 2, 35128 Padova, Italy; irene.condonato@studenti.it (I.C.); marcolovisotto1995@gmail.com (M.L.); paolo.simioni@unipd.it (P.S.); 2 Nephrology Unit, Department of Medicine, University of Padua, 35128 Padua, Italy; dorella.delprete@unipd.it (D.D.P.); francescakatiana.martino@aopd.veneto.it (F.K.M.);

**Keywords:** antiphospholipid antibodies, lupus anticoagulant, aPL-nephropathy, thrombotic angiopathy, thrombosis

## Abstract

Antiphospholipid syndrome (APS) can affect the kidneys, leading to renal artery and vein thrombosis, allograft loss following transplantation, and microvascular damage referred to as aPL-nephropathy (aPL-N). APL-N is a complex and frequently underdiagnosed condition characterized by an incomplete understanding of its etiopathogenesis and associated with unfavorable renal outcomes. The 2023 ACR/EULAR classification criteria for APS included aPL-N within the microvascular domain. The gold standard for aPL-N is the biopsy, revealing lesions associated with acute thrombotic microangiopathy and chronic vascular changes. Nevertheless, reluctance for biopsies due to anticoagulation and thrombocytopenia underscores the need for noninvasive diagnostics. Common clinical features include hypertension, microscopic hematuria, proteinuria, and renal insufficiency. Antiphospholipid antibodies seem crucial to kidney damage through thrombotic and inflammatory processes. Studies and experimental models of thrombotic microangiopathy lesions suggest the involvement of the complement cascade, tissue factor, and mammalian target of the rapamycin complex activation pathway. Currently, the management of aPL-N is based mainly on expert opinion, with limited evidence supporting the use of anticoagulants, leading to controversy in their application. Treatment may include heparin, intravenous immunoglobulin, plasma exchange, and targeted therapies tailored to aPL-N mechanisms. Future multicenter studies are essential to clarify their roles. The goal of this review is to inform clinicians and create a research agenda to address the unmet needs in diagnosing and managing APL-N.

## 1. Introduction

Antiphospholipid syndrome (APS) is a thrombo-inflammatory disorder with a complex and not fully understood pathogenesis. Recurrent thrombosis and pregnancy morbidity linked to the presence of circulating antiphospholipid antibodies (aPL) characterize APS. The aPL included in the recent ACR/EULAR 2023 classification criteria are anti-cardiolipin (aCL), anti-β2-glycoprotein I (anti-β2GPI), and lupus anticoagulant (LAC) [1,2]. With an estimated incidence of 50 patients per 10,000 person-years, APS predominantly affects young female adults aged 15 to 50 years, conferring significant morbidity [3]. The condition includes arterial or venous thrombosis, microvascular thrombosis, or recurrent thrombosis resistant to anticoagulants, potentially causing severe complications [4,5,6]. APS can affect various organs, including the kidneys, leading to a significantly higher rate of damage accrual and disability burden in this young population [7]. As recently reported [8], about one-third of aPL-positive patients develop kidney failure. The entire kidney vasculature may be involved in APS, such as renal arteries and veins, intrarenal arteries and arterioles, and glomerular capillaries [9]. Renal involvement in APS, particularly microvascular, is often underestimated due to the high-risk nature of renal biopsy, anticoagulant therapy, and potential thrombocytopenia. Indeed, in aPL-positive systemic lupus erythematosus (SLE) patients, kidney biopsies are significantly delayed compared to aPL-negative patients (134.4 ± 60.6 vs. 42.6 ± 60.1 months from SLE diagnosis without differences between aPL-positive SLE patients with or without APS criteria), risking misdiagnosis or delayed diagnosis [10,11]. Recently, the American College of Rheumatology (ACR)/European League Against Rheumatism (EULAR) 2023 APS classification criteria included aPL-nephropathy (aPL-N) in the microvascular domain [1]. This review explores the clinical phenotypes associated with renal involvement in APS and the potential pathogenetic mechanisms that underlie these phenomena. Its purpose is to inform clinicians and create a research agenda to address the unmet needs in diagnosing and managing aPL-N.

## 2. Epidemiology of Renal Involvement in APS

The prevalence of kidney involvement in APS remains poorly defined. Among the 1000 patients in the Euro-phospholipid registry [12], the prevalence of renal disease was 3%, of which 2% had glomerular thrombosis, 1% renal infarction, 0.5% renal arterial thrombosis, and 0.2% renal vein thrombosis. However, given the reluctance to perform biopsies, the true prevalence of aPL-N is unknown to date. In fact, in some cohorts that specifically evaluated aPL-N lesions, the prevalence ranged from 6% to 45% [10,13,14]. A recent systematic review of the literature on biopsy-proven aPL-N revealed a prevalence of 28.6% [8]. In CAPS, acute renal involvement reaches 71%, as reported in the CAPS-Registry [15].

## 3. Pathophysiology of Renal Involvement in APS

**Figure 1:**
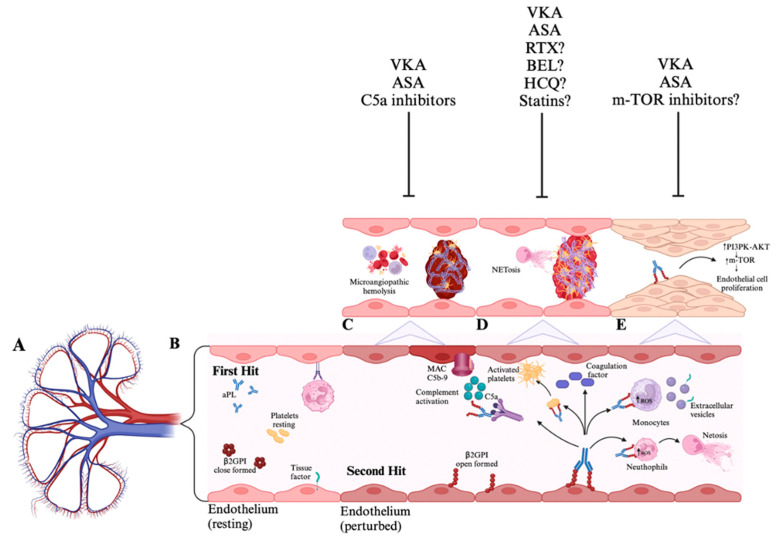
Potential pathogenesis of antiphospholipid antibody nephropathy and possible pathway inhibitors. (**A**) Kidney vasculature. (**B**) The pathophysiology of APS is explained by the “two-hits hypothesis”, where aPL, particularly anti-β2GPI, acts as the initial catalyst in creating a prothrombotic state. However, this alone is insufficient to cause thrombosis. A second hit, such as stasis or inflammation, results in endothelial cell damage that disrupts natural anticoagulant systems, promoting thrombosis. The endothelial cell dysfunction mediated by aPL activates endothelial cells, platelets, monocytes, and neutrophils, promoting the release of neutrophil extracellular traps and tissue factor expression and activation of the complement cascade. (**C**) The complement-mediated tissue factor plays a critical role in the pathogenesis of thrombotic microangiopathy in APS; thus, treatment with C5a inhibitors, alongside anticoagulation and/or antiplatelet therapies, may reduce kidney injury. (**D**) The activation of neutrophils by aPL leads to the release of neutrophil extracellular traps and tissue factor expression, fostering thrombus development and vascular injury. Therefore, anticoagulation and/or anti-platelet therapy may sufficiently address vascular injury. Sometimes, treatment with immunosuppressants, such as rituximab, belimumab, hydroxychloroquine, or statins that inhibit tissue factor expression, could be beneficial. (**E**) The aPL antibodies interact with endothelial cells via the mTOR pathway, whereby the activation of the mTOR complex stimulates the growth and proliferation of endothelial cells, contributing to the chronic vasculopathy seen in chronic APL-N lesions. Consequently, treatment with mTOR inhibitors combined with anticoagulation and/or antiplatelet therapy may help alleviate kidney injury. APS: antiphospholipid syndrome; aPL: antiphospholipid antibodies; mammalian target of rapamycin: mTOR; VKA: vitamin K-antagonist; ASA: aspirin; RTX: rituximab; BEL: belimumab; HCQ: hydroxychloroquine; NETosis: neutrophil extracellular traps; MAC: membrane attack complex; β2GPI: β2 glycoprotein I. Created with https://BioRender.com (accessed on 7 January 2025).

The pathogenesis of renal APS involvement is complex and not fully understood. The strong link between aPL and aPL-N suggests these antibodies play a crucial role in renal injury. Clinical presentations of macrovascular and microvascular involvement, with acute and chronic lesions, indicate various underlying mechanisms (Figure 1). Macrovascular involvement follows the “two-hit model of APS”, where anti-β2GPI initiates a prothrombotic state, further worsened by stasis or inflammation systems [2]. APL interacts with various cell types, including platelets, granulocytes, and endothelial cells, promoting intravascular coagulation by cross-reacting with plasma proteins like tissue factor (TF) pathway inhibitors. Furthermore, aPL triggers monocytes’ TF release, leading to factor Xa release and thrombin formation generation [16]. Anti-β2GPI antibodies bind targets, aggregating and forming immune complexes on platelets and endothelial cells, enhancing prothrombotic activity by activating Apolipoprotein E receptor 2 (ApoER2) [16,17]. Murine models recognized this mechanism as an essential trigger of APS [17]. Interestingly, mice that have anti-β2GPI antibodies and are deficient in the ApoER2 (−/−) gene showed reduced thrombotic events compared to wild-type mice. Immune complexes attach to endothelial cells, prompting the expression of adhesion molecules like selectins and integrins. This mechanism activates endothelial cells and enhances TF expression [2,16,17]. Molecular activations from immune complexes activate the complement system and promote neutrophil extracellular trap release (NETs) [2]. Additionally, the complement-mediated TF seems to contribute to the development of thrombotic microangiopathy (TMA) in APS [18]. A key factor in aPL-mediated thrombosis, both in ex vivo and in vitro studies [19,20,21], was the complement cascade activation. Research by Seshan et al. [22] showed that mouse-derived IgG aPL and human-IgG aPL can induce glomerular lesions typical of TMA in mice. They observed increased fibrin, TF, and complement C3 deposits in the glomeruli of mice given both mouse and human aPL, highlighting these factors’ role in TMA pathogenesis [22]. Furthermore, neutralizing CD59 in a renal TMA mouse model resulted in more C5b-9 formation in glomeruli, increased platelet and fibrin deposition, more severe endothelial injury, and reduced renal function compared with controls [23]. A clinical study of 42 renal tissue samples confirmed TMA due to various conditions (eight SLE/APS). It found C4d and C5b-9 in 88.1% and 78.6% of TMA cases, respectively, noting distinct staining patterns in the renal vasculature across conditions [24]. C4d staining and microthrombi coexist in aPL-positive SLE biopsy sample patients [23,24,25].

Chronic vascular lesions display different histopathological changes, with fibrous intimal hyperplasia (FIH) being the most common. While the exact cause of these lesions remains unclear, it is probably not just due to thrombotic injury. Canaud et al. [26] suggested that chronic activation of the mammalian target of the rapamycin complex (mTOR) pathway might play a role in these patients, which is associated with the emergence of chronic vasculopathy lesions. Their research showed no vascular lesion recurrence and reduced proliferation in the renal transplant of aPL-N patients treated with sirolimus, confirmed by follow-up assessment biopsies. They also observed vascular activation of the mTOR pathway in autopsy specimens from CAPS patients [26].

## 4. Kidney Involvement in APS

**Figure 2:**
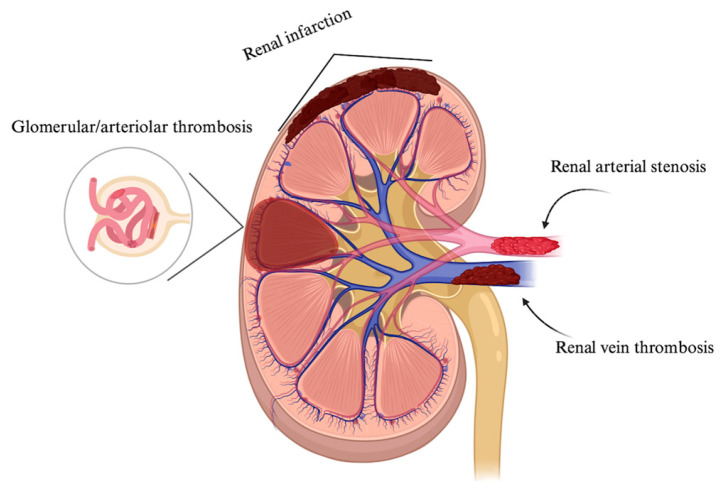
Involvement of kidney vasculature showing arterial, vein thrombosis, and microvascular lesions characterized by glomerular and arteriolar thrombosis. Created with https://BioRender.com (accessed on 7 January 2025).

APS renal involvement includes three groups based on location, namely renal artery lesions, intrarenal vascular lesions, and renal vein thrombosis, as shown in Figure 2. Renal artery lesions and vein thrombosis represent macrovascular involvement, acknowledged in the Sapporo classification criteria [27], while intrarenal lesions indicating microvascular involvement were recently added to the 2023 ACR/EULAR classification criteria [1].

### 4.1. Macrovascular Involvement

#### 4.1.1. Renal Arterial Stenosis

Though uncommon in APS, thrombosis of the renal artery and branches leads to renal infarction. First described in the early 90s [28], it can present as unilateral or bilateral thrombosis from in situ thrombosis or arterial embolism, possibly from cardiac chambers or proximal arteries. Renal artery disease may manifest as renal infarction, ischemic acute renal failure, or slowly progressive chronic renal failure [29,30,31]. Key clinical signs of renal artery stenosis include severe new-onset hypertension or the worsening of existing hypertension, often accompanied by flank pain and macrohematuria [30,31,32]. Sangle et al. [32] studied 77 patients with aPL and uncontrolled hypertension, 91 hypertension clinic attendees, and 92 potential kidney donors. MRI renal angiography diagnosed renal artery stenosis in 26% of the study sample, notably higher than in other groups. Consequently, APS patients with uncontrolled hypertension should raise suspicions of renal artery thrombosis, and young patients with unexplained renal artery thrombosis should undergo testing for aPL.

To evaluate renal artery stenosis, renal artery Doppler ultrasound is preferred. Although renal angiography has the highest accuracy, contrast-enhanced CT and MRI are less invasive alternatives with similar diagnostic value performance [33].

Renal artery involvement in APS has two key radiological traits that set it apart from fibrodysplasia and atherosclerosis: (i) it primarily affects the proximal segment of renal arteries and (ii) it presents as a smooth, well-defined lesion, usually resulting in non-critical stenosis [32]. Growing interest exists in contrast-enhanced ultrasound for assessing perfusion abnormalities in patients with renal impairment infarction [34].

#### 4.1.2. Renal Vein Thrombosis

Renal vein thrombosis can occur unilaterally or bilaterally in individuals with APS, being more common in those also diagnosed with SLE. The main clinical sign linked to renal vein thrombosis is nephrotic-range proteinuria, and in some cases, bilateral involvement may lead to renal failure thrombosis [35,36,37,38]. Acute thrombosis may present with flank pain and, less frequently, hematuria. In APS patients, consider renal vein thrombosis with sudden nephrotic-range proteinuria, as it may complicate renal transplantation and affect outcomes. Doppler ultrasonography is the preferred diagnostic tool for showing an edematous kidney with reduced echogenicity, parenchymal disruption, and renal vein thrombus. Additionally, computed tomography venography may be performed. Be aware that intravenous line size, injection rate, hydration levels, and cardiac output significantly impact results. Renal vein thrombosis is a filling defect in post-contrast venous phase imaging administration [39].

### 4.2. Microvascular Involvement

#### 4.2.1. APL-Nephropathy

**Figure 3:**
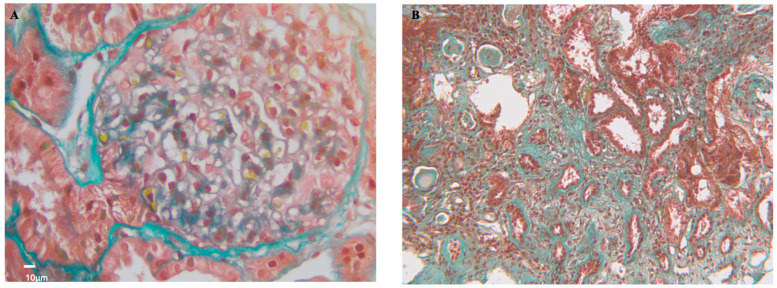
Antiphospholipid antibody nephropathy histology. (**A**) (Masson’s Trichrome stain 400×, scale bar 10 μm) The glomerular loops appear prominent and fill the urinary space. In some areas, the glomerular basement membranes have a fluffy appearance. Fragmented red blood cells are present in the lumen. The mesangial spaces appear expanded with pale-staining material (mesangiolysis). (**B**) (Masson’s Trichrome stain 200x, scale bar 10 μm) Tubulointerstitial compartment with sclerotic lesions, interstitial fibrosis, and tubular atrophy.

Identified in 1999, aPL-N is a small-vessel vasculopathy characterized by acute and chronic renal lesions. A seminal study by Nochy et al. [40] assessed renal biopsies of sixteen APS patients, revealing histological changes that clarify aPL-N diagnosis as follows: TMA, fibrous intimal hyperplasia, recanalizing thrombi, fibrous arterial occlusion, tubular pseudo-thyroidization, and focal cortical atrophy. TMA is a key histological finding in the acute form of aPL-N (Figure 3A), characterized by fibrin thrombi with fragmented blood cells in small vessels and glomeruli, along with subendothelial edema. The glomerular basement membrane may show a double-layered wrinkled appearance in advanced stages due to endothelial detachment and mesangial cell infiltration. Cellular inflammation and immune deposits seen in immunofluorescence are usually absent in primary cases of APS [40]. TMA lesions are more common in CAPS [41].

FIH is the most common chronic lesion linked to APS alongside focal cortical atrophy (FCA) and thyroidization (Figure 3B). FIH involves significant myofibroblastic proliferation in the intimal layer, sometimes displaying an “onion skin” appearance. Lumens may become obstructed by fibrous tissue, leading to recanalization and the development of endothelialized channels. FCA, resulting from prolonged reduced blood flow, affects the renal cortex beneath the capsule, causing contour depression and glomerular tissue retraction. Tubular thyroidization shows extensive atrophic tubules containing eosinophilic casts resembling thyroid tissue.

Both primary and secondary forms of APS show aPL-N. In 2002, Daugas et al. [42] evaluated renal biopsies from a substantial French cohort of patients with SLE, specifically investigating vascular lesions consistent with aPL-N. Their findings showed aPL-N in 63% of aPL-positive patients, and 78% of those had previous thrombosis or pregnancy issues. Other studies supported these results. Tektonidou et al. found aPL-N prevalence at 40% in aPL-positive SLE biopsy samples versus 4% in the aPL-negative cohort [43]. A strong association between aPL-N and arterial thrombosis APS and triple aPL positivity APS phenotypes has been described [8,14,43]. Furthermore, a notable link between aPL-N and livedo reticularis was reported [43].

Notably, patients with lupus nephritis and renal TMA have the worst outcomes among vascular damage manifestations, necessitating thorough evaluation assessment [44,45]. This observation is consistent with a longitudinal study involving 111 individuals diagnosed with lupus nephritis and monitored over 15 years, which revealed that patients who tested positive for aPL encountered the most detrimental renal outcomes [46]. An international multicenter study recently evaluated 123 kidney biopsies from patients who were aPL-positive [14]. Cluster analysis showed that renal TMA, acute or chronic, occurred more frequently in patients with thrombotic APS and triple aPL positivity. In contrast, other changes, like FIH, were less correlated with systemic thrombotic events. As such, a renal TMA cluster results in a worse renal prognosis.

APL-N can present with clinical signs like high blood pressure, microscopic hematuria, and varying levels of proteinuria, which may become severe, reaching the nephrotic range, alongside acute kidney injury or a gradual decline in chronic kidney disease. A recent meta-analysis [8] investigated risk factors associated with kidney failure in biopsy-proven aPL-N, identifying hypertension and triple aPL as strong predictors of adverse renal outcomes in aPL-N. Hypertension is the most common symptom of aPL-N. In a recent systematic review, over 70% of patients with biopsy-confirmed aPL-N were found affected with arterial hypertension compared to 48% in patients without aPL-N [8]. In the Nochy study [40], over 90% of participants experienced hypertension, attributed to excess renin production caused by vascular damage. Since hypertension often occurs in chronic kidney disease (CKD) patients, both as a cause and effect, the clinical presentation of aPL-N tends to be nonspecific. Other renal manifestations include mild proteinuria and a gradual decrease in kidney function. The combination of slowly worsening kidney function and mild proteinuria may lead clinicians to hesitate in recommending a kidney biopsy, particularly given the ongoing anticoagulant therapy and/or thrombocytopenia. However, these pathological changes, particularly TMA, are not pathognomonic of APS, making it challenging for clinicians to diagnose aPL-N. Consequently, although aPL-N is rare, it may go unrecognized and underdiagnosed.

As outlined in Table 1 and Table 2, the groundbreaking 2023 ACR/EULAR classification criteria include aPL-N within the microvascular domain, considering both suspected and definitive aPL-N based on the presence or absence of kidney biopsy [1]. Furthermore, the 2023 ACR/EULAR classification criteria from the Renal Pathology Subcommittee [47] provided a precise definition to homogenize the histopathological terminology, including the distinction of acute from chronic aPL-N lesions, as well as the distinction of thrombotic lesions in glomeruli from those in arteries/arterioles, and the separation of FIH from organized thrombi, as reported in Table 3. The goal is that the new 2023 ACR/EULAR APS classification criteria aPL-N definition, in conjunction with advancing technologies such as digital pathology, machine learning algorithms, spatial transcriptomics, and proteomics, will facilitate precise diagnosis. Furthermore, noninvasive techniques such as intravoxel incoherent motion diffusion-weighted imaging MRI, used to assess renal function in patients with immunoglobulin A nephropathy, may aid in diagnosing aPL-N in the future [48].

#### 4.2.2. Glomerular Lesions

In addition to the acute and chronic vascular lesions discussed earlier, aPL-N recognizes various glomerular lesions. In 2003, Fakhouri et al. [49] reported that 31% of the 29 patients studied exhibited “atypical” histological forms of aPL-N. Among these patients, they documented different glomerular lesions, including membranous nephropathy (two cases), minimal change in disease/focal segmental glomerulosclerosis (three cases), mesangial C3 nephropathy (three cases), and pauci-immune crescentic glomerulonephritis (one case). Sinico et al. [13], in a multicenter cohort study of 160 PAPS patients, evaluated the kidney biopsy of 10 patients with signs of renal injury; four patients displayed features indicative of aPL-N, four had membranous nephropathy, and two showed proliferative glomerulonephritis. A recent systematic literature review revealed that among 238 biopsy-proven aPL-N cases, 62.2% had diffuse proliferative glomerulonephritis, while 16.2% had membranous glomerulonephritis [8]. It is still uncertain whether a pathophysiological connection exists between these glomerulonephritis and aPL-N or if these lesions occurred independently and are mutually exclusive. There are ongoing studies to clarify these data.

## 5. Renal Transplantation in APS

Patients with aPL-N who undergo renal transplants face a high risk of thromboembolic events in the post-transplant period [50,51,52,53,54]. This risk is most significant in the first week, with renal vein thrombosis being the most common occurrence; intra- and extra-graft thrombosis can also occur [53,54,55]. APS progression in kidney transplant recipients is unpredictable, causing allograft thrombosis and potential loss. Complications include macrovascular thrombosis of renal vessels and TMA. A study of 19 adult kidney recipients with APS revealed a significantly lower 15-year renal allograft survival rate compared to controls (*p* = 0.0009), especially in those diagnosed with APS for a later transplant [55].

The estimated prevalence of aPL in renal transplant recipients is around 3% [51]. Patients with aPL-positive pre-transplantation had higher chronic vascular scores and faster mGFR decline after one year compared to aPL-negative patients (mean 49.1 ± 18.4 vs. 54.4 ± 19.4, *p* = 0.04); LA is the most common aPL antibody. Emerging evidence about non-criteria aPL is being reported. Serrano et al. [56] found that renal transplant recipients with IgA anti-B2GP1 face higher thrombosis risk if they develop IgA-β2-glycoprotein I immunocomplexes. There are no data yet regarding anti-phosphatidylserine/prothrombin antibodies (aPS/PT), known to be associated with thrombosis and LA in APS [57,58]. Within our cohort, four out of five APS patients who underwent renal transplants tested positive for aPS/PT antibodies (personal communication).

## 6. Management of Renal Involvement in APS

**Figure 4:**
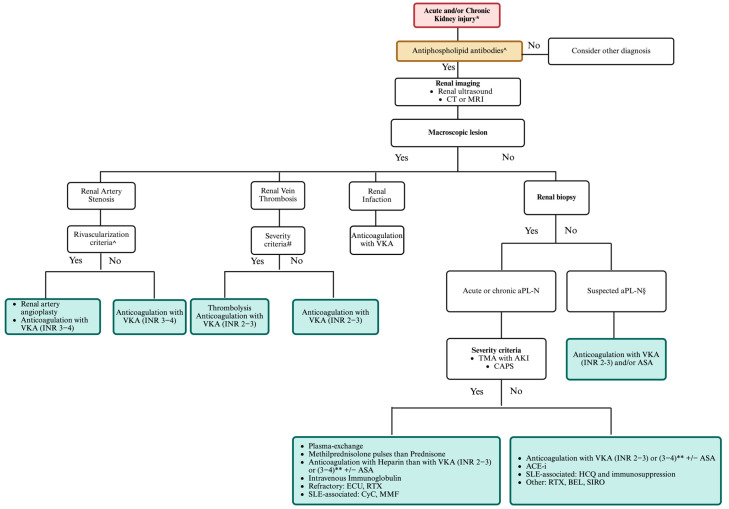
Management algorithm of kidney damage in antiphospholipid syndrome. * New onset/refractory hypertension, glomerular proteinuria/hematuria, acute kidney failure, chronic or end-stage renal disease of unknown origin, history of SLE. ^ Medium–high titer anticardiolipin antibodies and/or anti-β2glycoprotein I IgG/IgM isotype and/or lupus anticoagulant confirmed ≥12 weeks apart. # allograft, acute kidney injury, single kidney. § Unexplained persistent: (a) new onset/deterioration hypertension; (b) proteinuria ≥ 0.5 g in 24 h urine specimen or protein/creatinine ratio ≥ 0.5 mg/mg (50 mg/mmol); (c) acute renal failure; (d) glomerular microscopic hematuria. ** INR 2–3 if associated with venous thrombosis, INR 3–4 if associated with arterial thrombosis. Created with https://BioRender.com.

Patients with macrovascular involvement who meet the 2023 ACR/EULAR criteria should use vitamin K antagonists (VKAs), targeting an INR of 2–3 for venous thrombosis or 3–4 for arterial or recurrent thrombosis, as shown in Figure 4. Patients with renal artery stenosis should receive prompt evaluation for angioplasty. Sangle et al. [59] studied 14 patients who underwent angioplasty, noting restenosis in those inadequately anticoagulated (mean INR < 2.5).

There is no consensus on managing aPL-N due to insufficient robust studies [29]. VKAs are the recommended standard of care [60] for patients meeting aPL-N histological criteria and 2023 ACR/EULAR classification criteria. Although the evidence for combining antiplatelets and VKAs is limited, clinical observations indicate that this approach may improve renal outcomes (personal experience). Preliminary data from our cohort of patients with microvascular involvement showed that in 4/4 (100%) patients with timely diagnosis of aPL-N and early treatment with combined anti-platelet drugs and VKAs led to improvement in renal function (personal unpublished data). The management strategy for aPL-N patients who do not meet classification remains ambiguous. Patients with concurrent lupus nephritis typically receive hydroxychloroquine and immunosuppressive therapies [61,62], while hypertension and proteinuria associated with aPL-N are treated using angiotensin system inhibitors [61,62]. Notably, the use of direct oral anticoagulants (DOACs) in aPL-N patients has not been specifically studied. DOACs, although not explicitly studied for aPL-N, their use in APS, particularly in patients with high-risk triple-positive APS or in those who experience arterial or recurrent thrombotic events, as indicated by the alarming results of the TRAPS study [63,64], are not recommended. A recent meta-analysis [65] of four open-label randomized clinical trials involving 472 patients with APS (median time of control arm in the therapeutic range: 60%) found a 5-fold increased risk of subsequent arterial thrombosis (OR 5.43; 95% CI 1.87–15.79). At the same time, no difference regarding the risk of venous thrombosis in the DOAC group compared to the warfarin one was observed [65]. This evidence suggests that DOACs may be ineffective for aPL-N patients, particularly given the noted association between arterial thrombosis and aPL-N. The current literature lacks robust studies assessing the effects of anticoagulation on renal outcomes.

In treating severe or refractory cases of antiphospholipid syndrome, heparin and treatments such as intravenous immunoglobulin or plasma exchange may be considered as alternative therapies [66,67].

There is a growing interest in targeted therapies, including B-cell-directed therapies, complement inhibitors, tissue factor inhibitors, and mTOR pathway inhibitors. Nonetheless, comprehensive prospective multicenter studies are crucial to understanding their effectiveness. A pilot open-label phase II trial specifically explored the use of rituximab, which showed promise as two patients with aPL-N reported partial responses after receiving multiple doses [68]. Additionally, multiple case reports have pointed to success with anti-CD20 treatments in patients experiencing non-criteria manifestations of APS, like severe thrombocytopenia and skin ulcers [69,70].

Belimumab, a BAFF antagonist, has been applied in cases of primary APS with reported clinical improvements, allowing patients to discontinue glucocorticosteroids [71]. A phase II trial is presently assessing the safety and effectiveness of belimumab for managing refractory or non-criteria antiphospholipid syndrome symptoms, fostering hope for enhanced treatment options [72].

Eculizumab—a humanized recombinant monoclonal antibody—binds to C5, shows efficacy in managing refractory cases of CAPS [73], post-kidney transplant TMA [74,75], and lupus nephritis with TMA [76]. There is ongoing research including data from the CAPS registry [77] which could shed light on the efficacy of these advanced therapies for patients grappling with the complexities of aPL-N and associated conditions.

Inhibiting the mTOR pathway presents a promising treatment for aPL-N, significantly reducing vascular proliferation in kidney biopsies and recurrence of vascular lesions in transplant recipients. Research by Canaud et al. [26] indicates that 70% of patients on mTOR inhibition had a functioning allograft a decade post-transplant compared to just 11% of untreated patients. Notably, the treatment’s efficacy was independent of anticoagulation therapy.

Recent studies have uncovered a type I interferon signature in primary APS, revealing that endothelial progenitor cells from affected patients show diminished differentiation abilities, suggesting that neutralizing type I interferon could improve their dysfunction [78,79,80]. Recently, a murine model of aPL-N revealed increased IFN signature expression levels in APS mice kidneys [81]. The novel tyrosine kinase 2 inhibitor reversed pathological vascular changes in mice kidneys and decreased fibrin and C3 deposition, suggesting a role of anti-interferon antibodies as a new therapeutic strategy to reduce vascular damage associated with APS.

Additionally, statins like pravastatin appear beneficial by down-regulating TF synthesis, preventing glomerular injury and TMA associated with aPL-N [22]. Sodium–glucose cotransporter inhibitors (iSGLT-2) have transformed chronic kidney disease (CKD) management but have not been tested for aPL-N due to exclusions in recent trials [82,83]. Despite this, iSGLT-2 may provide renal protection, as it influences the mTOR pathway. Given the thrombotic nature of aPL-N, further clinical investigations specifically focused on this patient group are essential to validate the therapeutic potential of iSGLT-2 in this context. Overall, advancing these treatments could significantly improve outcomes for aPL-N patients.

## 7. Unmet Needs and Future Strategies in Antiphospholipid Antibody Nephropathy

Despite progress in understanding APS, significant unmet needs remain in diagnosing and managing aPL-N. The 2023 ACR/EULAR classification criteria Renal Pathology Subcommittee’s efforts in defining aPL-N have resulted in only minor changes compared to two decades ago [47]. Diagnosing aPL-N depends on histological changes that are not exclusive to APS. However, this initiative provides a detailed list of histological variations to standardize pathological reporting of aPL-N [47]. Advanced technologies like digital pathology and machine learning can identify early endothelial cell injury in glomeruli and blood vessels, improving diagnoses and differentiating TMA causes. Although renal biopsy is the gold standard, performing renal biopsy in APS patients is challenging due to increased thrombotic risk, anticoagulant use, and frequent thrombocytopenia. We lack universally accepted guidelines for anticoagulation management or platelet count thresholds for aPL-N biopsy. Nonetheless, extrapolating from existing expert consensus and KDIGO and ASH guidelines [84,85,86] for high-risk biopsy situations, a platelet count above 50.000/µL is generally advised as a safe threshold to proceed with percutaneous renal biopsy. Anticoagulation therapy (particularly VKA and DOACs) should be withheld with bridging to low-molecular-weight heparin when possible, restarting anticoagulation 48–72 h after biopsy if no bleeding complications occur. When significant thrombocytopenia (platelets < 50.000/µL) or high bleeding risk precludes standard percutaneous biopsy, alternative approaches such as transjugular renal biopsy might considered. Indeed, transjugular renal biopsy provides a safer alternative, minimizing bleeding risk and allowing for prompt anticoagulation restart and should thus be considered in patients with severe thrombocytopenia or who cannot safely interrupt anticoagulation. Multidisciplinary discussion (nephrologists, rheumatologists, hematologists, vascular medicine physicians, and radiologists) is recommended for individualized decisions. Challenges in performing kidney biopsies highlight the urgent need for noninvasive diagnostic alternatives [8,10]. Recent advancements in noninvasive renal assessment, such as diffusion-weighted imaging, hold promise for patients with immunoglobulin A and diabetic nephropathy, paving the way for innovative diagnostics approaches [87,88].

Currently, we do not have biomarkers capable of identifying aPL-N in its early stages, particularly for differentiating it from other kidney issues such as lupus nephritis or hypertensive nephropathy. Recent studies of kidney tissue transcriptome data have shown increased expression of complement, interferon, and NET genes in APS kidneys, indicating potential biomarkers for therapeutic targets [89]. Earlier studies have elucidated the role of complement activation in obstetric APS and, more recently, in thrombotic APS as well [90]. Furthermore, plasma levels of C5a and the complement activation product C5b-9 were found to be significantly elevated in patients with CAPS and those suffering from refractory thrombotic APS [91,92]. The involvement of neutrophils and NETs in thrombosis has been recognized, highlighting the connection between C5a and neutrophil activation during pregnancy complications, as well as their interferogenic roles in both APS and SLE [93].

Current guidelines focus on APS broadly, neglecting nephropathy as a distinct condition [61]. Uncertainty persists regarding effective treatment for aPL-N, including anticoagulants, immunosuppressants, and combination therapies. Patient responses vary significantly, highlighting the need for more research on factors influencing treatment outcomes. Urgent long-term studies are needed to assess aPL-N progression and treatment effectiveness. Identifying risk factors for CKD or end-stage renal disease in aPL-N patients is a critical unmet need.

## 8. Conclusions

APL-N is a complex, often underdiagnosed condition with unclear etiopathogenesis and poor renal outcomes. Improving our understanding of its mechanisms, identifying risk factors, and using noninvasive diagnostic methods could lead to earlier diagnoses and new treatment options, enhancing patient prognosis.

## Figures and Tables

**Table 1:** The 2023 ACR/EULAR definition for antiphospholipid syndrome.

Entry Criteria		
≥1 Clinical Criterion Plus ≥ 1 aPL Test		
(aPL Positive Within Three Years from the Clinical Criterion)		
Clinical Domain		Score
**Domain 1**	**Venous Thromboembolism**	
	with VTE high-risk profile	1
	without VTE high-risk profile	3
**Domain 2**	**Arterial Thrombosis**	
	with AT high-risk profile	2
	without AT high-risk profile	4
**Domain 3**	**Microvascular**	
	Suspected *	2
	Livedo racemose	
	Livedoid vasculopathy	
	Acute/chronic aPL-N	
	Pulmonary hemorrhage	
	Established §	5
	Livedoid vasculopathy	
	Acute/chronic aPL-N	
	Pulmonary hemorrhage	
	Myocardial disease	
	Adrenal hemorrhage	
**Domain 4**	**Obstetric**	
	≥ 3 consecutive pre-fetal (<10 WG) and/or early fetal (<16 WG)	1
	Fetal death (≥16 WG) without PEC/PI	1
	Severe PEC or Severe PI (<34 WG)	3
	Severe PEC and Severe PI (<34 WG)	4
**Domain 5**	**Cardiac Valve**	
	Thickening	2
	Vegetation	4
**Domain 6**	**Hematology**	
	Thrombocytopenia (20–130 × 109/L)	2
**Laboratory Domain**		
**Domain 7**	**Lupus anticoagulant test**	
	One-time positive	1
	Persistent positive	5
**Domain 8**	**Anti-cardiolipin and anti-** **β2-glycoprotein I ^**	
	Moderate–high positive IgM aCL and/or anti-β2GPI	1
	Moderate positive IgG aCL and/or anti-β2GPI	4
	High positive IgG aCL or anti-β2GPI	5
	High positive IgG aCL and anti-β-2GPI	7

* Diagnosed by exam and/or laboratory tests and/or imaging. § Diagnosed by imaging and/or pathology. ^ Solid phase assay. aPL: antiphospholipid antibodies; VTE: venous thromboembolism; AT: arterial thrombosis; aPL-N: antiphospholipid antibody nephropathy; WG: week of gestation; PEC: pre-eclampsia; PI: placental insufficiency; aCL: anti-cardiolipin antibodies; anti-β2GPI: anti-β2 glycoprotein I antibodies; IgM: immunoglobulin M; IgG: immunoglobulin G.

**Table 2:** The 2023 ACR/EULAR definition for antiphospholipid antibody nephropathy.

aPL-N	Definition
Suspected aPL-N *	New-onset hypertension or deterioration of previously well-controlled hypertension
	Proteinuria ≥ 0.5 g in 24 h urine specimen or protein/creatinine ratio ≥0.5 mg/mg (50 mg/mmol)
	Acute renal failure
	Glomerular microscopic hematuria
**Established aPL-N ^**	Acute renal vascular or glomerular thrombotic microangiopathy
	Chronic renal vascular or glomerular lesions

aPL-N: antiphospholipid antibody nephropathy; gm: gramme; mg: milligrams; mmlo: micromoles. * By physical examination or laboratory tests. ^ By pathology.

**Table 3:** The 2023 ACR/EULAR histology definition for antiphospholipid antibody nephropathy.

Type of Lesion	Definition
**Acute aPL-N**	Fibrin thrombi in arterioles or glomeruli without inflammatory cells or immune complexes
**Chronic aPL-N**	Organized arterial or arteriolar microthrombi with or without recanalization
	Fibrous and fibrocellular arterial or arteriolar occlusions
	Cortical atrophy with or without thyroidization
	Fibrous intimal hyperplasia
	Organized glomerular thrombi

aPL-N: antiphospholipid antibody nephropathy.

## Data Availability

All data generated or analyzed during this study are included in the respective cited articles.
